# Infection and Vaccination-Induced Tick-Borne Encephalitis Virus IgG Antibody Prevalence in the Austrian Federal State of Upper Austria, a High-Risk Region for TBEV

**DOI:** 10.3390/epidemiologia7020035

**Published:** 2026-03-02

**Authors:** Gerhard Dobler, Susanne Süßner, Anne B. Schindler, Philipp Girl, Johannes Borde

**Affiliations:** 1 Institut für Mikrobiologie der Bundeswehr, Neuherbergstrasse 11, 80937 München, Germany; 2 Department of Parasitology, University of Hohenheim, Emil-Wolff-Strasse 34, 70599 Stuttgart, Germany; 3 Division of Infectious Diseases and Tropical Medicine, Medical Center of the University of LMU Munich, Leopoldstrasse 5, 80802 Munich, Germany; 4 Austrian Red Cross, Blood Transfusion Service of Upper Austria, Krankenhausstrasse 7, 4010 Linz, Austria; 5 Praxis Prof. Dr. J. Borde & Kollegen, Am Marktplatz 8, 77704 Oberkirch, Germany; 6 Division of Infectious Diseases, Department of Medicine, University Medical Center Freiburg, i.Br. Hugstetter Str. 55, 79106 Freiburg im Breisgau, Germany; 7 Bacteriology and Mycology, Institute for Infectious Diseases and Zoonoses, Department of Veterinary Sciences, Faculty of Veterinary Medicine, Ludwig Maximilian University of Munich, Sonnenstrasse 24, 85764 Oberschleissheim, Germany

**Keywords:** tick-borne encephalitis, anti-TBEV-NS1-IgG, ELISA, epidemiology

## Abstract

Background/Objectives: Tick-borne encephalitis (TBE) is the most important tick-borne viral central nervous system (CNS) infection in Europe and Asia. Since the introduction of a vaccine in Austria in the late 1970s, sero-epidemiological studies on the true incidence of tick-borne encephalitis virus (TBEV) infection in the population have been difficult, because it was not possible to distinguish between vaccine- and infection-induced antibodies. The goal of our study has been to analyze the sero-epidemiology of TBEV infections, vaccination protection rate, and manifestation index of the disease in the region of interest. Methods: Applying a newly developed anti-TBEV-NS1-IgG assay and the neutralization test, the protection and infection rates in blood donors of the Austrian Federal State of Upper Austria.It is one of the first areas in Austria, where the TBEV vaccine had been rolled out and broadly used. Samples from blood donors of all districts of the Federal State of Upper Austria were screened for anti-TBEV-IgG. Positive sera were differentiated for infection- and vaccine-induced antibodies. The results were matched with donor age, gender, and geographical origin. Results: 2162 samples were analyzed. A total of 87.0% of the blood donors tested showed anti-TBEV-IgG related to past TBEV vaccination. Within the unvaccinated group, a total of 13.3% of male and 9.9% of female blood donors exhibited anti-TBEV-NS1-IgG, indicating a past TBEV infection. The anti-TBE-NS1-IgG seroprevalence was determined at 74/100,000 for the whole population and at 594/100,000 in the non-vaccinated population. The manifestation index (MI) was calculated at 2.8%. The MI is defined as the probability or percentage of infected individuals who develop clinical symptoms of a disease. Conclusions: Our data provide evidence of a continuing high risk of TBEV infection in the Federal state of Upper Austria. The non-vaccinated population has an eightfold higher infection prevalence compared to the whole population. The MI of TBEV for severe infection seems lower as detailed in previous reports.

## 1. Introduction

Tick-borne encephalitis (TBE) is an infectious disease, mainly of the central nervous system (CNS), which is caused by the tick-borne encephalitis virus (TBEV), now according to modern virological taxonomy, *Orthoflavivirus encephalitidis* [1]. To date, at least 5 different subtypes, with regard to newer genetic information, even seven subtypes of TBEV may be distinguished. Among them, the European subtype (TBEV-EU), the Siberian subtype (TBEV-Sib), and the Far Eastern subtype (TBEV-FE) are of main human importance, while the Baikalian subtype (TBEV-BAIK) and the Himalayan subtype (TBEV-Him) are of local relevance. Some more genetically different TBEV strains may turn out to be separate lineages or subtypes (e.g., strain 178-79, strain Obskaya, strain Sallandse) [2,3]. The TBEV is mainly transmitted by hard ticks, in Europe predominantly *Ixodes ricinus*, in Asia *Ixodes persulcatus* and *Ixodes ovatus*, but hard ticks of the genera *Dermacentor* and *Haemaphysalis* may play a role in local transmission as well [4]. Another way of transmission is the alimentary infection by unpasteurized milk and raw milk products [5].

Tick-borne encephalitis is the most important tick-borne virus infection and is distributed in Europe and Asia. As an emerging infection, TBE is increasingly reported from previously unaffected areas and is also re-emerging in many well-known endemic countries in Europe. In 2021, the presence of TBEV was shown for the first time outside of Europe and Asia in Tunisia, Africa [6]. During the last few years, an increase in notified human TBEV infections has been reported in many countries throughout Europe. It is estimated that more than 10,000 cases may occur every year in Europe and Asia, although many of these infections may remain undiagnosed or unrecognized [7].

Since the detection of TBEV in Europe in 1948 in the former Czechoslovak Republic [8], this virus has been tracked in many European countries, and its medical impact has been recognized. Notably, the increasing medical importance of the disease was underscored in the 1960s, when Austria reported the highest TBE morbidity in Europe. Although the clinical entity was already described decades ago in 1931 by the Austrian specialist for internal medicine, Hans Schneider, who named the disease “epidemic acute serous meningitis”, which finally turned out to be TBE [9]. The medical awareness of TBE in Austria increased, especially during the 1950s and 1960s [10]. Consequently, Kunz et al., at the Institute of Virology at the University of Vienna, developed the first European TBEV vaccine in the 1970s, and large vaccination campaigns were launched, mainly in school children, in the 1980s to combat the disease [11,12].

Before the introduction of the TBEV vaccine, the annual number of diagnosed human cases in Austria ranged between 300 and 700. After the broad use of the TBEV vaccine, cases decreased to a range between 40 and 130 in the years between 1995 and 2017, with an absolute minimum number of 41 cases in the year 1999 [13]. Since starting the country-wide vaccination campaign, the vaccination coverage reached >80% in the Austrian population this correlated indirectly with the decrease in human cases. Today’s vaccine coverage, determined by vaccination sales and marketing interviews, still results in excellent country-wide vaccine coverage rates of 80 to 85%. In Europe/Austria, there are two licensed TBEV vaccines available, FSME-Immun^®^ (Pfizer) using the TBEV strain Neudoerfl and Encepur^®^ (Bavarian Nordic) using the TBEV strain K23. A detailed description of the inactivated whole virus vaccines is beyond the scope of this introduction. It should be kept in mind that in Austria, the FSME-Immun^®^ (Pfizer) vaccine has been used predominantly.

**Figure 1:**
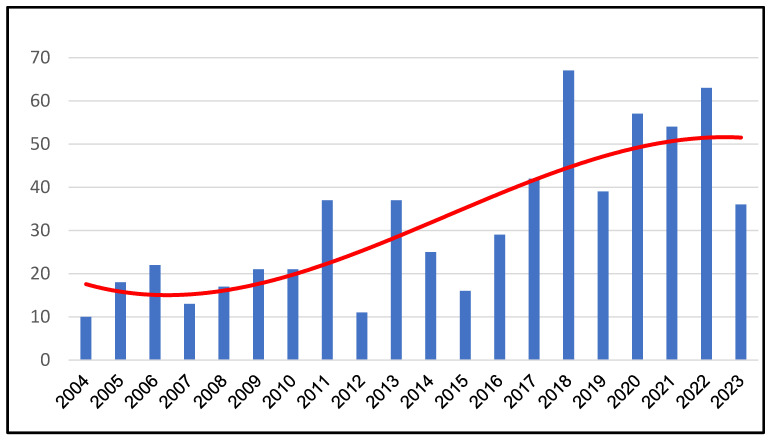
Reported human TBE cases (blue bars) 2004 to 2023 in the Federal State of Upper Austria and trend of TBE cases (red line) (adapted from data of the Annual Reports of Reportable Infectious Diseases in Austria).

However, since 2018, the number of annually reported human TBE cases has been increasing again from 108 to 217 in the years 2018 to 2024 and has never been below the number of 100 annual reported cases (Figure 1) [14]. The reasons for this newly increasing trend of human TBE cases in Europe are obscure. Seroprevalence studies had been difficult or impossible to conduct as a differentiation of vaccine- and infection-induced antibodies has not been feasible. Therefore, the true infection prevalence rate in the population has not been analyzed for more than 40 years. Only a small proportion of TBEV infection cases may manifest enough clinical symptoms to cause patients and physicians to diagnose the disease [15]. Furthermore, the recent occurrence of West Nile virus (WNV), mainly in eastern parts of Austria, a former highly endemic area for TBE, the increasing travel activities of the population with increasing numbers of imported flavivirus infections (e.g., dengue fever, between 2015–2024 there were 123 notified dengue fever cases in Upper Austria) and travel-vaccine induced flavivirus antibodies (e.g., yellow fever vaccine) may complicate antibody studies due to cross reactivities among the flavivirus group.

In 2018, Albinsson et al. developed an assay, based on the detection of antibodies against the non-structural protein NS1 of TBEV, which allows differentiation between vaccine- and infection-induced antibodies against TBEV [16]. In 2020, Girl et al. developed a similar assay, based on an ELISA platform [17]. Although initially developed for diagnostic purposes, this test has also demonstrated its utility in sero-epidemiological studies [18]. It could be shown in individual serum samples that anti-TBEV-NS1-IgG could be detected up to 20 years after confirmed TBEV infection (Dobler, personal data).

Using the ELISA assay of Girl et al., a study was initiated, testing sera of blood donors of the Austrian federal state of Upper Austria for IgG antibodies against TBEV and differentiating the IgG antibodies for infection and vaccination. According to the Austrian Federal Ministry of Work, Social, Health, Care and Consumer Protection for Upper Austria the reported TBE cases more than doubled from an average of 21 cases (2004 to 2015) to 49 (2016 to 2024) (Annual Reports of Reportable Infectious Diseases in Austria, https://www.sozialministerium.gv.at/Themen/Gesundheit/Uebertragbare-Krankheiten/ accessed on 20 July 2025) (Figure 1). This increase in human cases raises questions about the underlying causes, such as a decline in vaccination coverage, missing vaccine boosters, a decreasing efficacy of the vaccine with increasing numbers of vaccine failures, or a shift of TBEV into new endemic areas due to climate change, with a low-vaccinated population at risk [19].

## 2. Materials and Methods

### 2.1. Samples and Sites

**Figure 2:**
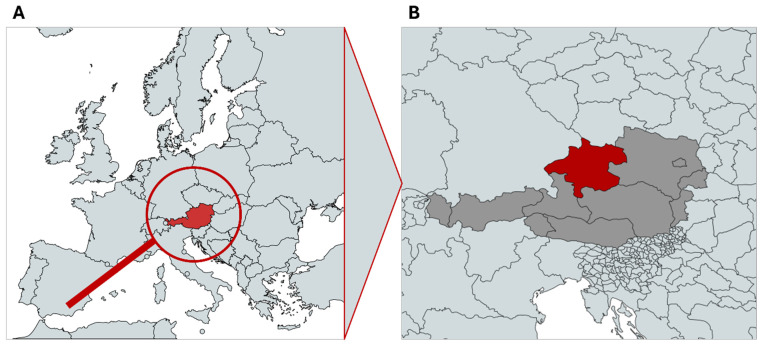
(**A**) Overview of the study location. (**B**) Map of Austria (grey) with the Federal State of Upper Austria (red). Created with MapChart (https://www.mapchart.net/index.html accessed on 1 December 2025).

Blood samples of blood donors were sampled by the Upper Austrian Red Cross Blood Donor Service from 13 March 2023 to 7 April 2023 (Figure 2). Blood samples were accessed for research purposes on 11 April 2023. They were kept at +4 °C until serum was separated by centrifugation. Serum was stored at +4 °C until testing. For each blood sample, the place of residence (zip code), age, and gender were collected for further epidemiological analysis. All samples were anonymized for the testing institution.

### 2.2. Ethical Approval

The study was conducted according to the Declaration of Helsinki. An ethical approval was given by the Ethical Committee of the Kepler University Hospital of Linz, Austria, under the number 1017/2023 and date of approval 13 March 2023.

### 2.3. Serological Testing

The serological testing was done as described in a former study of Siller et al. [20]. Sera were screened by an anti-TBEV-IgG ELISA (Fa. Euroimmun, Luebeck, Germany) according to the instructions of the manufacturer. Anti-TBEV-IgG positive sera were confirmed by the TBEV micro-neutralization test (mNT) to exclude other flavivirus infections or vaccinations [21]. Sera with confirmed TBEV antibodies were further tested regarding anti-TBEV-NS1-IgG [17]. Anti-TBEV-NS1-IgG-positive sera were classified as past TBEV infection. Borderline sera were tested again. In case of a second positive or borderline result, the serum was classified as past TBEV infection. In case of discrepant results (one positive/borderline, one negative result), the serum was classified as negative.

### 2.4. Statistical Analysis

#### Epidemiological Data, Case Definition, Disease Incidence, and Manifestation Index

Data on the number of inhabitants were taken from the official homepage of Upper Austria. For prevalence and incidence analyses, we used an estimate of 1,540,078 inhabitants for Upper Austria.

The number of notified TBE cases was retrieved from the open-access database of the Annual Reports of Transmissible Diseases of the Austrian Federal Ministry of Work, Social, Health, Care, and Consumer Protection. In Austria, only hospitalized TBEV infections with CNS symptoms are notifiable. Non-hospitalized patients, patients with unspecific or mild TBE symptoms, are usually not reportable as TBE cases.

Based on other sero-epidemiological studies by Girl et al., individual case observations, and single case reports, it is increasingly conclusive that a subclinical TBEV infection after vaccination, usually (except vaccine failures), will not induce the formation of anti-TBEV-NS1-IgG. Otherwise, we would expect much higher anti-TBEV-NS1-IgG rates, notably in highly vaccine-protected populations like in Bavaria or Austria; however, this is not the case. A positive anti-TBEV-NS1-IgG either indicates past TBEV infection, in case of vaccinated people before TBE vaccination, or, rarely, may also indicate a clinically significant vaccination breakthrough infection. This means that most of the anti-TBEV-NS1-IgG positive patients should have been non-vaccinated when acquiring their TBEV infection. It must be kept in mind that we estimate the persistence of anti-TBEV-NS1-IgG for 20 years (as demonstrated previously in historic serum samples by Girl et al.). Therefore, the number of non-vaccinated was calculated as the sum of TBEV-IgG-negative plus anti-TBEV-NS1-IgG-positive.

The manifestation index (MI) of TBE was calculated using the portion of the estimated total number of infections in the non-immune portion of the population and the number of reported TBE cases in Upper Austria from 2004 to 2023. The index showed the proportion of reported cases per total estimated cases multiplied by 100.

## 3. Results

**Figure 3:**
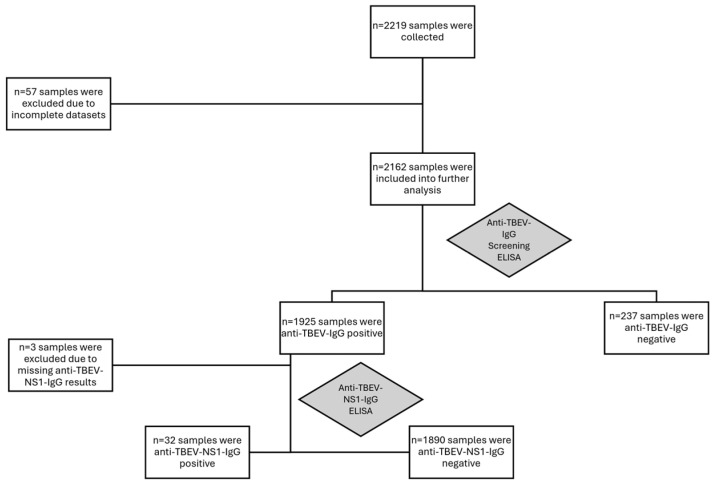
Schematic results of the seroprevalence study.

**Figure 4:**
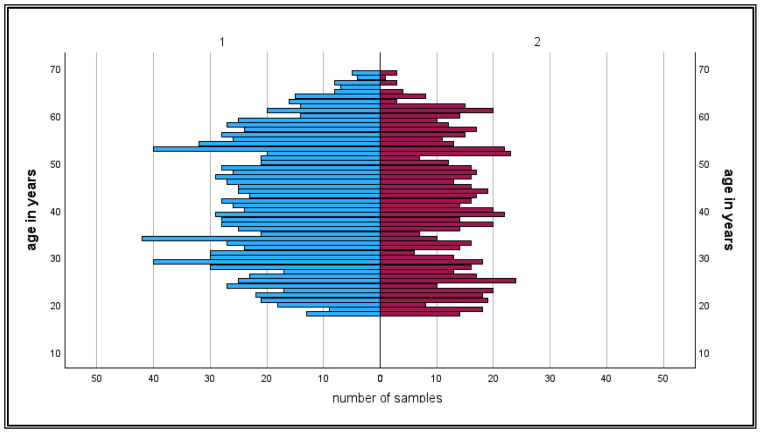
Age-dependence of TBE IgG positive blood donors. Male donors are denoted as 1 (blue bars) and female donors as 2 (pink bars).

A total of 2219 sera were collected (see for an overview Table 1). Due to missing data or incorrect place of residence (tourists donating blood in Upper Austria), 57 sera had to be excluded from the analysis. A total of 2162 sera met all inclusion criteria for the analysis. Of all included sera, 1925/2162 (~89%) reacted positively or borderline in the anti-TBEV-IgG screening ELISA, indicating either TBEV vaccination, TBEV infection, or flavivirus cross reaction (Figure 3 and Figure 4). All screening positive sera were confirmed by mNT with a titer of ≥20. Therefore, no cross-reacting flavivirus IgG was detected in the study population.

**Figure 5:**
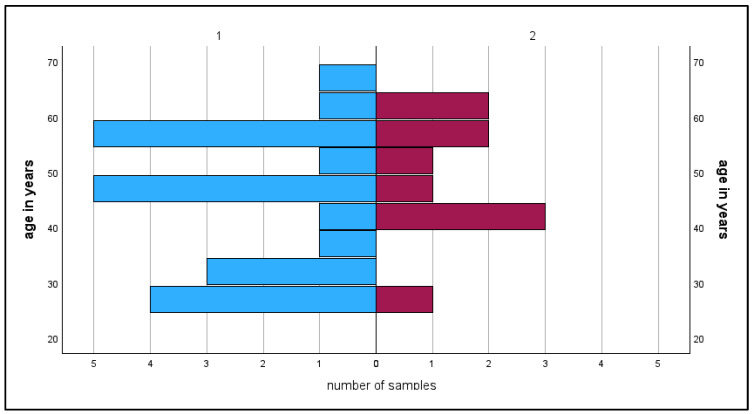
Age-dependence of TBEV-infected blood donors. Male donors are denoted as 1 (blue bars) and female donors as 2 (pink bars).

All anti-TBEV-IgG positive were tested in the anti-TBEV-NS1-IgG assay. A total of 32 sera reacted positively or repeatedly borderline, so that 32/2162 of all blood donors showed evidence of a past TBEV infection (Figure 5). If these were subtracted from the 1922 anti-TBEV-IgG positive samples, a total of 1890/2162 (~87%) of the blood donors showed evidence of vaccination against TBEV. A total of 237/2162 (~11%) samples exhibited no evidence of either TBEV infection or TBE vaccination. As there is now good evidence that anti-TBEV-NS1-IgG is only formed by infection before vaccination or after rare breakthrough infections, we can assume that the anti-TBEV-NS1-IgG-positive sera must have been non-vaccinated at the time of infection. Therefore, we calculate the total number of non-infected persons in the study at 269. A total of 269/2162 (~12%) of blood donors were not vaccinated against TBEV at all or at the time of TBEV infection. As vaccinated persons usually will not be susceptible to serologically provable TBEV infection (shown by anti-TBEV-NS1-IgG seroconversion), the TBEV infection prevalence was 32/269 (~12%). As anti-TBEV-NS1-IgG persists up to 20 years after acute TBEV infection, these results indicate that non-vaccinated inhabitants of Upper Austria may have a risk of infection of 12%/20 years or 0.6% per year living in this TBEV-endemic high-risk area.

### 3.1. Gender-Specific Vaccination Rates and Anti-TBEV-NS1 IgG Prevalences

A total of 1350/2162 (62.4%) samples were taken from male donors. Of these, 1206/1350 samples showed positive results in the anti-TBEV-IgG screening assay. 22/1206 sera reacted positively in the anti-TBEV-NS1-IgG assay. These data result in a vaccination rate of ~88% for males and an infection rate of 1.6% for the total male population and ~13% of the unvaccinated male study population.

812/2162 samples (37.6%) derived from female donors. A total of 719/812 samples reacted positively in the screening anti-TBEV-IgG ELISA. Of these, 10 sera reacted with anti-TBEV-NS1-IgG, indicating past TBEV infection. A total of 719/812 sera (~88%) of female blood donors showed evidence of vaccination, while 10/812 samples (1.2%) of all tested female samples indicated a past TBEV infection, and the proportion of TBEV infection in the non-vaccinated female blood donor population was 10/103 (~10%).

### 3.2. Age-Specific Vaccination Rate and Anti-TBEV-NS1 IgG Prevalence

**Figure 6:**
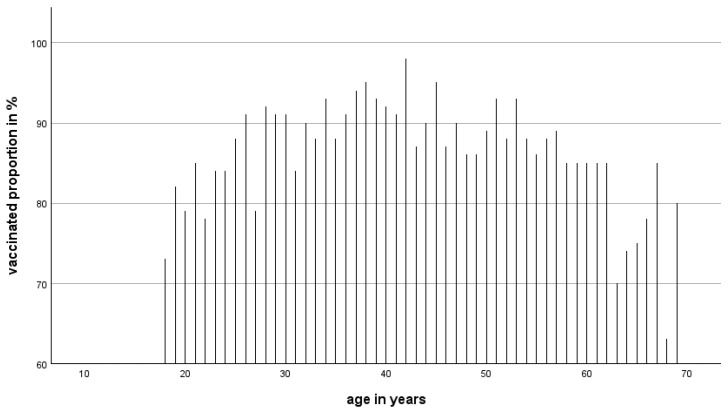
Age-specific vaccination rate and anti-TBEV-NS1 IgG prevalence.

The rate of unvaccinated persons (Figure 6) decreases with age from ~21% in the age group 18–19 years to below 10% in the age group 50–54 years and then increases to >20% in the higher age groups. In contrast, the infection rates are highest in the middle age groups 40–44 years, 45–50 years, and 55–59 years (>20% each) and decrease again to below 10% in the higher age groups. The age-dependent results are presented in Figure 6.

### 3.3. District-Specific Vaccination Rates and Anti-TBEV-IgG Prevalence

**Figure 7:**
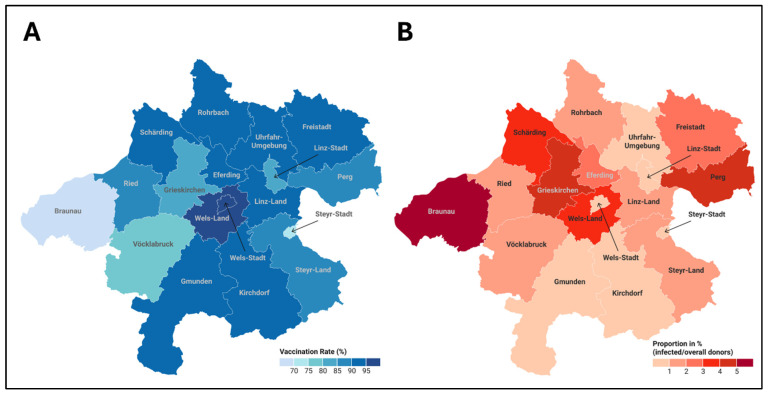
(**A**) Proportion of vaccinated participants (in %) across districts, illustrating regional differences in vaccination coverage. (**B**) Proportion of participants who tested positive for infection (in %) across districts, highlighting the geographic distribution of infection rates. Created in Datawrapper.de.

Upper Austria is politically divided into 18 districts. We received samples of all 18 districts; however, with varying sample numbers per district, ranging from 7 samples (city of Wels) up to 293 samples (district of Voecklabruck). The vaccination rates ranged from 68.6% (district of Braunau) to 100% (city of Wels). The proportion of non-vaccinated blood donors ranged from ~0% (city of Wels) up to 31.4% (district of Braunau). The proportion of anti-TBEV-NS1 IgG positives per non-vaccinated ranged from 0% (cities of Linz, Steyr, Wels; districts of Gmunden, Kirchdorf, Urfahr-Umgebung) up to ~30% (districts of Schaerding, Freistadt, Eferding) (Table 1) and (Figure 7).

### 3.4. Calculated Incidence Rate and Manifestation Index (MI) of TBEV Infections in Upper Austria

We found a total of 32 anti-TBEV-NS1-IgG positives in 2162 blood donors. Assuming that the risk of infection is similar in blood donors and non-donors, the prevalence for the whole population results in a total of ~23,000 human infections for the whole population of Upper Austria in 20 years. The calculated anti-TBEV-NS1-IgG positive incidence using only the susceptible and unvaccinated population results in an incidence of 594/100,000 inhabitants per year (using the complete population as an at-risk population, the incidence is 74/100,000 inhabitants per year). The incidence of notified TBEV cases, using the published dataset 2004–2023, in Upper Austria is 2.1/100,000 inhabitants per year. The manifestation index (MI) is at 2.8% when using the total population as the at-risk population, regardless of being vaccinated or not. When using the unvaccinated proportion as the susceptible at-risk population, the MI is 0.35%.

## 4. Discussion

Tick-borne encephalitis was since the 1960s the most important endemic viral disease of the CNS with increasing case numbers reaching a maximum of 677 cases in 1979 [22]. With the universal introduction of the TBE vaccine in Austria, the reported cases decreased within 10 years after implementation to below 100 reported cases in most of the years between 1990 and 2015 [13]. A comparison with the neighboring Czech Republic clearly showed the effect of the vaccination campaign in Austria [23]. Since 2016, an increase in TBE cases has been reported in many central European countries, as well as in Austria. The reasons for this increasing trend are not understood and are often discussed as natural fluctuation due to environmental and ecological reasons, including climate change [24,25].

The reasons for this increase in reported human cases are obscure. The reporting system has not changed during the last two decades. Also, no changes or improvements to diagnostic assays were implemented. The risk and prevalence rate of infection in the population has been unknown since the introduction of the vaccination, as TBEV antibodies could not be differentiated between vaccine- and infection-induced. TBE only clinically manifests in about 10% [15]. As these data are some 60 years old, it is unknown if the pathogenicity of the circulating TBEV strains may have changed, and whether the manifestation index increased or decreased. Recent studies show that the manifestation index may be as low as 2–5% [20,21], arguing for an even higher number of sub-clinical infections. It is a matter of discussion whether the environmental changes regarding the climatic changes may increase the risk of infection and, therefore, cause higher infection rates. However, the interactions of the different associated partners in the natural transmission cycle are complex and therefore difficult to evaluate [26]. Furthermore, the extension of spatial and temporal transmission cycles may be involved, leading to longer transmission of TBEV in larger geographic areas. Another reason to be discussed is the waning protection due to decreasing vaccination activities in the population.

According to current data (commercial interviews of a population sample by a vaccine manufacturer and the Institute of Virology, Medical University of Vienna), the vaccination rate in the Austrian population reached a maximum level of 88% in 2004 to 2006, since then a small decrease of the vaccination rate to about 80% was reported for the years 2019 to 2022 [13]. Using the assay for the detection of anti-TBEV-NS1-IgG, it became possible to differentiate between vaccine- and infection- induced antibodies. This assay was used successfully in three epidemiological studies [18,20,27]. We used this test to perform a study in blood donors from the Austrian Federal State of Austria to clarify the reasons for the doubling of human TBE cases during the last 10 years.

The Federal State of Upper Austria is well known as a TBE high-endemic region, and some of the very first vaccination field tests were conducted in the Buermoos Area in Upper Austria in forest workers and farmers (Kunz, pers. communication) before the introduction and universal recommendation of the TBE vaccination. Therefore, a high vaccination rate might be expected. Upper Austria is also a geographically interesting area as its altitude ranges from the Danube River and Inn River lowlands to high mountainous areas (Alps). Therefore, a geographical expansion into higher altitudes might be seen in the study.

In our study, we found an overall protection rate of 86.2% (1901/2167). This rate is somewhat higher than in a similar study in the Federal States of Tirol and Vorarlberg. The age-dependent vaccination rates in our data show that the protection rate was highest in the 35 to 55-year age group, with around 90% (Table 1). This is higher than the protection rate in the Austrian Federal States of Tirol and Vorarlberg (80.6%) [20]. In a study 30 years ago, the vaccination rate was estimated at 75% in Upper Austria [28].

The age group of 18–24 years and the age group of 60 to 69 years showed significantly lower protection rates of about 80%. This could indicate that a proportion of the younger blood donors did not get vaccinated and that the older portion of blood donors may have waning neutralizing antibodies. A similar distribution of vaccine-induced antibodies was also described in a Swiss study [29]. These results may have implications for the basic vaccination rates in younger age groups and maybe in the booster rates in the older age groups, which both might be increased to provide protection in these age groups, especially sensitive and vulnerable to TBEV infection [30,31].

Regarding the infection rates indicated by the presence of anti-TBEV-NS1-IgG, a total of 32/2167 (1.48%) reacted positively (Table 1). This rate seems low in comparison with findings before the introduction of vaccination, where the antibody prevalence rates in endemic countries ranged from 0 to 40%, depending on the location and population group [32,33,34]. Comparing with recent epidemiological data of anti-TBEV-NS1-IgG prevalence rates in the Austrian Federal States of Tirol and Vorarlberg, we found a sero-prevalence rate of 2.7% [20]. In southwestern Germany, the sero-epidemiological results resulted in an anti-TBEV-NS1-IgG rate of 5.6% in the whole population [18]. Another research group used the anti-TBEV-NS1-IgG assay in blood donors in Denmark and found a prevalence rate of 0.1% [35]. In a Swiss study, anti-TBEV-NS1-IgG was studied in vaccinated and non-vaccinated Swiss individuals. The seroprevalence rates were 0.8% in the non-vaccinated and 5.0% in the vaccinated group [29].

Our data now indicate that vaccinees who are boosted by natural infection will not develop anti-TBEV-NS1-IgG (except vaccine breakthrough TBEV infections). We calculated the susceptible persons for TBEV infection with the formation of anti-TBE-NS1-IgG as the sero-negatives and the anti-TBEV-NS1-IgG positives (at the time point of infection, also anti-TBEV-IgG negative). Using this calculation, the age-specific infection rates ranged from 0% (18–25 years age group) to 24% (55–59 years age group) and then declined again. In the age group > 60 years, the anti-TBEV-NS1-IgG rate declined again. This decline may be explainable with the disappearance of the anti-TBEV-NS1-IgG after 15 to 20 years. In the age groups of 35–40 years and 50–55 years, the rates are significantly lower. So far, there is no explanation available for this phenomenon except that this may be a statistical issue.

In accordance with the published data, we found a vaccination rate of 87.7% in the tested blood donors. The rates varied in the different districts from 68.9% (District of Braunau) to 100% (City of Wels). The results are somewhat higher than the published 81% for the whole of Austria (2022). This might be due to the selected population of blood donors tested in the study. It is known that blood donors usually have some differences in their health status in comparison to the general population (healthy donor effect) [36]. Another reason for the discrepancy might be that we did not test the vaccination rate but the protection rate measured by neutralizing antibodies. Former studies showed that the protection rate might be higher than the vaccination rates, especially in populations with lower vaccination rates [21]. However, we think that this might not have had much impact in this high vaccination rate.

The infection rates in the particular districts range from 0% (rural districts of Kirchdorf, Gmunden, Urfahr-Umgebung; city districts of Wels, Linz, Steyr) to 33.3% (district of Schaerding). As the rural districts of Kirchdorf and Gmunden are in the southern part of Upper Austria including the mountains and also the district of Voecklabruck shows a very low infection prevalence of 3%, these data clearly indicate that TBEV so far has not entered the mountainous areas of Austria and causes a relevant part of infections which might be responsible for the increase of human cases seen in the past years.

The highest seroprevalence rates with ~30% of positive anti-TBEV-NS1-IgG each are found in the districts of Schaerding, Freistadt, and Eferding in the northern central part of Upper Austria. The high seroprevalence rate in the district of Schaerding correlates well with data from the adjacent districts of Passau and Freyung-Grafenau in Germany [27]. The district of Rohrbach neighbors the southern Bohemian region of the Czech Republic, one of the regions in Europe with the highest TBE incidence [37]. Rohrbach shows a medium-high prevalence rate of 12.5%. However, this district exhibits a vaccination rate of higher than 90%, and therefore, the susceptible population might be low. Surprisingly, the adjacent district of Urfahr-Umgebung had an infection rate of 0%. However, this district exhibited the highest protection rate of all rural districts with 93.5%. This might be one reason, maybe, besides some statistical bias in the selection of blood donors.

Regarding the manifestation index of clinical overt TBE infections with CNS manifestation, we found an index of 2.8%. This correlates very well with earlier data of the district of Ortenaukreis (southwestern Germany) and the Austrian Federal states of Tirol and Vorarlberg, where very similar manifestation indices were determined [18,20,27]. This result is interesting as the definition of reportable TBEV infections is somewhat different in Austria and Germany. While in Austria, only CNS-manifest TBEV infection is reportable; in Germany, any detection of positive IgM and IgG is reportable as acute TBEV infection. Therefore, we expected a somewhat lower manifestation index in comparison to the data in Germany. However, new data from a research group at the Robert-Koch Institute showed that most patients in Germany (84%) reported at least some symptoms of the CNS when the anamnestic questions addressed this symptom complex [38].

## 5. Conclusions

The sero-prevalence study in Upper Austria revealed a high protection status in the population, with decreasing rates in the younger and older age groups. Therefore, this high vaccination rate might not be the reason for the increasing reported TBE cases over the last few years. Also, no evidence of TBEV infection was found in the mountainous areas. The highest infection rates were found in the well-known high-risk areas along the frontier to the Czech Republic and in the central wooded areas of the lowlands of the Federal State, as already described during the last decades. According to these results, we conclude that the increase in human cases during the last few years is not due to an expansion into the mountainous areas of the Federal State, but it seems that the natural risk of infection increased in the traditional well-known risk areas. The reasons for this increase in infection remain unclear, but might include changes in human behavior and/or in the circulation and transmission cycle of the TBE virus due to environmental changes. The clinical manifestation rate of TBEV infection seems to be lower than described so far. According to our results, only 2.8% of the infected individuals manifest symptoms of the CNS. This rate is in accordance with recent epidemiological studies using the detection of anti-TBEV-NS1-IgG.

## Figures and Tables

**Table 1:** General Overview, counties of Upper Austria with inhabitants, study population, and results (^1^ TBE vaccinated donors: anti-TBEV-IgG positive and anti-TBEV-NS1-IgG negative; ^2^ Donors with past TBEV infection: anti-TBEV-IgG positive and anti-TBEV-NS1-IgG positive; ^3^ Donors at risk: Susceptible or previously susceptible population for a TBEV infection with anti-TBEV-NS1-IgG seroconversion, i.e., summation of unvaccinated donors plus infected donors).

County	Inhabitants	Number *n* of Included Samples (% from Overall Samples)	TBEV Vaccinated Donors ^1^ (Vaccination Rate in %)	Donors with Past TBEV Infection ^2^	Proportion in % (Infected/Overall Donors)	Proportion in % (Infected/Donors at Risk) ^3^
**Braunau**	110,571	85 (3.9)	57 (67)	5	6	18
**Eferding**	33,884	88 (4.1)	82 (93)	2	2	33
**Freistadt**	68,326	134 (6.2)	124 (93)	3	2	30
**Gmunden**	103,155	267 (12.3)	246 (92)	0	0	0
**Grieskirchen**	66,750	46 (2.1)	37 (80)	2	4	22
**Kirchdorf**	58,199	77 (3.6)	70 (91)	0	0	0
**Linz**	213,557	69 (3.2)	58 (84)	0	0	0
**Linz-Land**	157,151	106 (4.9)	95 (90)	1	1	9
**Perg**	70,516	123 (5.7)	108 (88)	5	4	33
**Ried/Innkreis**	63,577	203 (9.4)	174 (86)	2	1	7
**Rohrbach**	62,461	162 (7.5)	146 (90)	2	1	13
**Schaerding**	58,433	182 (8.4)	165 (91)	5	3	29
**Steyr-Land**	62,613	160 (7.4)	142 (89)	2	1	11
**Steyr-Stadt**	37,917	25 (1.2)	21 (84)	0	0	0
**Urfahr-Umgebung**	88,388	103 (4.8)	96 (93)	0	0	0
**Voecklabruck**	141,988	293 (13.6)	231 (79)	2	1	3
**Wels-Land**	77,110	32 (1.5)	31 (97)	1	3	100
**Wels-Stadt**	65,482	7 (0.3)	7 (100)	0	0	0
**Total**	**1,540,078**	**2162 (100)**	**1890 (87)**	**32**	**1**	**12**

## Data Availability

Data are available on reasonable requests to the corresponding author due to concerns regarding the anonymity of the donors from sites with a very low sample size.
